# SimRNAweb v2.0: a web server for RNA folding simulations and 3D structure modeling, with optional restraints and enhanced analysis of folding trajectories

**DOI:** 10.1093/nar/gkae356

**Published:** 2024-05-13

**Authors:** S Naeim Moafinejad, Belisa R H de Aquino, Michał J Boniecki, Iswarya P N Pandaranadar Jeyeram, Grigory Nikolaev, Marcin Magnus, Masoud Amiri Farsani, Nagendar Goud Badepally, Tomasz K Wirecki, Filip Stefaniak, Janusz M Bujnicki

**Affiliations:** Laboratory of Bioinformatics and Protein Engineering, International Institute of Molecular and Cell Biology in Warsaw, ul. Ks. Trojdena 4, PL-02-109 Warsaw, Poland; Laboratory of Bioinformatics and Protein Engineering, International Institute of Molecular and Cell Biology in Warsaw, ul. Ks. Trojdena 4, PL-02-109 Warsaw, Poland; Laboratory of Bioinformatics and Protein Engineering, International Institute of Molecular and Cell Biology in Warsaw, ul. Ks. Trojdena 4, PL-02-109 Warsaw, Poland; Laboratory of Bioinformatics and Protein Engineering, International Institute of Molecular and Cell Biology in Warsaw, ul. Ks. Trojdena 4, PL-02-109 Warsaw, Poland; Laboratory of Bioinformatics and Protein Engineering, International Institute of Molecular and Cell Biology in Warsaw, ul. Ks. Trojdena 4, PL-02-109 Warsaw, Poland; Department of Molecular and Cellular Biology, Harvard University, 52 Oxford St, Cambridge, MA 02138, USA; Laboratory of Bioinformatics and Protein Engineering, International Institute of Molecular and Cell Biology in Warsaw, ul. Ks. Trojdena 4, PL-02-109 Warsaw, Poland; Laboratory of Bioinformatics and Protein Engineering, International Institute of Molecular and Cell Biology in Warsaw, ul. Ks. Trojdena 4, PL-02-109 Warsaw, Poland; Laboratory of Bioinformatics and Protein Engineering, International Institute of Molecular and Cell Biology in Warsaw, ul. Ks. Trojdena 4, PL-02-109 Warsaw, Poland; Laboratory of Bioinformatics and Protein Engineering, International Institute of Molecular and Cell Biology in Warsaw, ul. Ks. Trojdena 4, PL-02-109 Warsaw, Poland; Laboratory of Bioinformatics and Protein Engineering, International Institute of Molecular and Cell Biology in Warsaw, ul. Ks. Trojdena 4, PL-02-109 Warsaw, Poland

## Abstract

Research on ribonucleic acid (RNA) structures and functions benefits from easy-to-use tools for computational prediction and analyses of RNA three-dimensional (3D) structure. The SimRNAweb server version 2.0 offers an enhanced, user-friendly platform for RNA 3D structure prediction and analysis of RNA folding trajectories based on the SimRNA method. SimRNA employs a coarse-grained model, Monte Carlo sampling and statistical potentials to explore RNA conformational space, optionally guided by spatial restraints. Recognized for its accuracy in RNA 3D structure prediction in RNA-Puzzles and CASP competitions, SimRNA is particularly useful for incorporating restraints based on experimental data. The new server version introduces performance optimizations and extends user control over simulations and the processing of results. It allows the application of various hard and soft restraints, accommodating alternative structures involving canonical and noncanonical base pairs and unpaired residues, while also integrating data from chemical probing methods. Enhanced features include an improved analysis of folding trajectories, offering advanced clustering options and multiple analyses of the generated trajectories. These updates provide comprehensive tools for detailed RNA structure analysis. SimRNAweb v2.0 significantly broadens the scope of RNA modeling, emphasizing flexibility and user-defined parameter control. The web server is available at https://genesilico.pl/SimRNAweb.

## Graphical Abstract

**Graphical Abstract:**
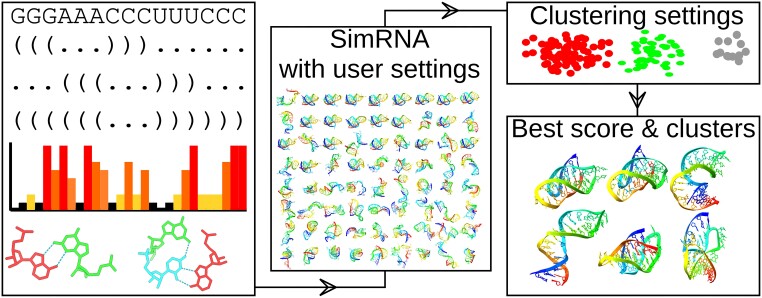

## Introduction

Ribonucleic acid (RNA) is essential in many biological processes, including carrying and transmitting genetic information, regulating gene expression and catalyzing biochemical reactions. The function of RNAs often relies on their ability to form specific three-dimensional (3D) structures. However, experimentally determining RNA 3D structures presents considerable challenges, resulting in a deficiency in structural data for many RNA sequences. In light of these challenges, computational prediction techniques have emerged as vital tools, supplementing experimental methods in the study of RNA. Computational methods have also been adapted to utilize the experimental data in the modeling process (1,2).

SimRNA, a method utilizing a coarse-grained representation and statistical potentials, has been developed to carry out RNA folding simulations and 3D structure prediction (3). SimRNA facilitates the initiation of analyses either from RNA sequence alone or from user-defined 3D structures. Additionally, it can integrate supplementary structural information, if provided, such as secondary structure restraints. The stand-alone version of SimRNA requires considerable effort from users for data processing, both to prepare the input information and to analyze the output data. To make the method more accessible, we introduced the SimRNAweb server, which simplified the use of SimRNA and made it available to broader users (2). This server has been particularly useful for researchers who are less familiar with command-line tools and to those without access to high-performance computing facilities. It is important to emphasize that compared to other web servers for RNA 3D structure prediction, such as RNAComposer (4), Vfold-Pipeline (5), FARFAR2 (6), trRosettaRNA (7) or DeepFoldRNA (8), SimRNAweb generates folding trajectories and can be used to study RNA conformational landscapes or carry out unfolding simulations for RNA stability analyses.

## Materials and methods

### Workflow implemented in SimRNAweb v2.0

The purpose of the SimRNAweb server is to manage the essential steps of preparing the necessary input parameters, executing SimRNA calculations with the required computational resources and generating the output. The server offers real-time simulation tracking and structure visualization. After the folding simulation, the server selects the highest-scoring conformations, performs their superposition and clustering, and generates all-atom models for the representatives of the largest clusters. It liberates users from the tasks of managing the simulation and processing the data, as these are comprehensively handled by the server. Users can access all output files, including logs and intermediate data, for further analysis.

The original SimRNAweb server provided users with very limited control over options, such as adjusting the simulation length and specifying the percentage of highest-scoring conformations for clustering. It also allowed users to enforce specific secondary structures and to input a list of atom–atom pairwise distance restraints. If a 3D structure was used as the starting point, users could ‘freeze’ selected residues to maintain their coordinates unchanged during the simulation. The output was processed with a default set of parameters, and once clustering and 3D model generation were completed, it was not possible to reanalyze the trajectory on the server with different parameters, without repeating the simulation.

In version 2.0 of the web server, the core SimRNA simulation engine remains largely the same as in the original version, including the coarse-grained RNA structure representation, the simulation scheme and the energy function. Due to space limitations in this article, we only describe the modifications and direct the reader to the article describing the original version of the web server (2) for a detailed description of the original simulation workflow, inputs and outputs. The handling of spatial restraints encoded in the input 3D structure files remains the same. The slope and well formats of pairwise distance restraints also remain the same. Since the publication of the original method (3), some minor bugs have been fixed, the program’s speed has been optimized and SimRNA has been updated to accommodate new types of restraints (described below). Minor adjustments were made to the log file format. The trajectory format now includes a header file, which clearly defines the parameters saved in the trajectory file. Major updates include a significant expansion of the input section and the addition of options for reanalyzing trajectories with enhanced clustering capabilities.

### Key additions in SimRNAweb v2.0

- Support for the PDBx/mmCIF format has been introduced, complementing the original PDB format for 3D structure coordinates.
- The capability to specify canonical base-pairing restraints for secondary structure now also accommodates noncanonical pairs.
- Users can now identify bases that are excluded from canonical pairing.
- Values of residue reactivities from chemical probing experiments, e.g. from SHAPE or DMS probing experiments (9), can be provided to be used as additional restraints influencing secondary structure formation in the simulation.
- Base-pair restraints, including both canonical and noncanonical, have been introduced in a new soft mode. In this mode, fulfilling a restraint grants a score bonus without imposing a penalty for violations, unlike the original hard restraints. Soft restraints allow for the exploration of alternative structures that may contain mutually incompatible pairs, thus facilitating the guidance of SimRNA simulations toward multiple potential structures.
- Simulation settings now offer control over the frequency of frame output, the temperature factor for the Monte Carlo simulation, the number of runs and the number of replicas. These enhancements enable users to conduct a broader range of simulations, including but not limited to replica-exchange Monte Carlo (REMC) simulations, single-replica folding and unfolding simulations. Previously, these features were only available in the stand-alone version of SimRNA.
- The capabilities for trajectory analysis in the new SimRNAweb server have been enhanced. The process initiates by computing an all-against-all root-mean-square deviation (RMSD) matrix, focusing on a user-specified percentage of the best-scored frames in the trajectory. Typically, RMSD evaluations are based on the pairwise superposition of frame coordinates. We have now introduced a feature where RMSD can be calculated directly from the coordinates in the trajectory, bypassing the superposition step. This enhancement is invaluable for simulations with partially ‘frozen’ (immobilized) RNA molecule models (e.g. for the refinement of existing models of RNA 3D structure), as it allows the RMSD to exclusively reflect the dynamics of the mobile regions relative to the frozen core.
- We have broadened the clustering functionalities to incorporate two novel methods for identifying clusters among the highest-scored frames in a SimRNA trajectory. Previously, the server only offered Option D (the default setting in SimRNA), which identifies a frame that attracts the maximal count of best-scored frames within a specified clustering threshold. This frame is designated as the cluster representative, the set of frames is extracted as the cluster and this process is recursively applied to the remaining best-scored frames to delineate up to five largest clusters. The newly integrated Option C stipulates that every cluster member must maintain an interframe RMSD below a user-defined threshold, with the cluster’s representative being the frame that minimizes the cumulative RMSD to all other frames. This option is useful for the identification of clusters that are relatively tight in terms of conformational variability. Additionally, Option E has been introduced, where initially the highest-scored frame is identified, and all frames with an RMSD below a specified threshold relative to this frame are grouped and extracted, and this method is iteratively applied to the remaining frames.
- In terms of output, users have the ability to reanalyze trajectories and reapply clustering with different parameters without the need to rerun the simulation. It is now possible to save the resulting models (the best-scored model and the representatives of up to five top clusters) in the PDBx/mmCIF format.

### New way of handling base-pairing restraints in SimRNAweb v2.0

When information on base pairs is available from experimental analyses or computational predictions using other methods, it can be input as restraints for SimRNA simulations. For cases involving only secondary structure, it can be provided as a single line in dot-bracket format. When pseudoknots are present, multiple lines indicating non-nested helices should be used. In the original SimRNAweb server, these restraints were applied with a slope-type penalty based on the distances between corresponding atoms in the nucleotides involved. In this approach, now referred to as hard restraints, a penalty is added to the SimRNA scoring function whenever the residues meant to pair are too distant or incorrectly oriented, and the RNA secondary structure is strictly enforced by predefined base pairs. The use of hard-type secondary structures is advised when the user is certain of a single specific pairing pattern in the RNA structure. However, such restraints are not suitable for defining alternative (mutually exclusive) pairs, as restraining one residue to several different partners in a hard manner causes SimRNA to attempt to bring all pairing partners together, resulting in overlapping pairs and artificially distorted structures.

Besides slope-type penalties, distance restraints in SimRNA also offer a well-type bonus, as described in the original article about the SimRNA method (3). If a restraint is met, the scoring function rewards the conformation. Conversely, if a restraint is violated, no penalty is applied. This allows for the definition of multiple different (even mutually incompatible) well-type restraints. In SimRNAweb v2.0, we have now introduced the use of such soft restraints on secondary structure. Soft-type secondary structure restraints are recommended for cases where the user is unsure about the predicted secondary structures or wishes to investigate various alternative structures (e.g. as observed in riboswitches).

In SimRNAweb v2.0, we have also introduced the capability to provide restraints on noncanonical pairs. Technically, they are handled similarly to canonical pairs, i.e. a specific base-pair restraint is translated into a set of three atom–atom pairwise distance restraints. Noncanonical base-pairing restraints should be provided in a format expressed as ‘chain *i*;residue *i* number;residue *i* type;chain *j*;residue *j* number;residue *j* type;pair type’ according to the Leontis–Westhof nomenclature (10) For example, A;8;A;A;21;G;HSt indicates that in chain A, nucleotide number 8, which is adenine, forms a noncanonical base pair with another nucleotide in chain A, nucleotide number 21, identified as guanine, in a Hoogsteen–sugar configuration with the *trans* orientation of the glycosidic bonds. The restraints on noncanonical pairs can be defined as either hard or soft, with soft restraints allowing for alternative pairings for the same residue.

In addition to guiding SimRNA simulations to form specific base pairs, SimRNAweb v2.0 now also enables specifying residues that should not form pairs. Currently, it is possible to discourage residues from forming pairs involving their Watson–Crick edges by using the symbol ‘x’ along with the dot-bracket notation for secondary structure.

Last but not least, in SimRNAweb v2.0, we enabled the use of restraints derived from chemical probing data. This type of restraint involves the reactivity of each nucleotide separated by commas ‘,’ and spaces must be used to separate different chains. The reactivity values must be between 0 and 1, with values below 0 interpreted as 0 and values above 1 as 1. If a reactivity is not assigned to a nucleotide, SimRNA skips that nucleotide in calculating the chemical probing restraint score. During each simulation step, the program predicts the current secondary structure and creates an array of 0 or 1 values, with 0 representing bases involved in canonical base pairing, indicated by ‘(’ or ‘)’, and 1 representing bases not involved in canonical base pairing, indicated by ‘.’. The final score for this type of restraint is the squared error between the predicted secondary structure from SimRNA and the chemical probing data provided by the user. SimRNAweb server uses a similar data format as tour RNAProbe web server for RNA secondary structure prediction (11) and can be used as a follow-up to generate a 3D structural model for an RNA sequence with secondary structure predicted by RNAProbe.

## Results

### Example applications with the use of the new options of the SimRNA 2.0 server

#### Simulations with soft restraints on alternative secondary structures

**Figure 1.**
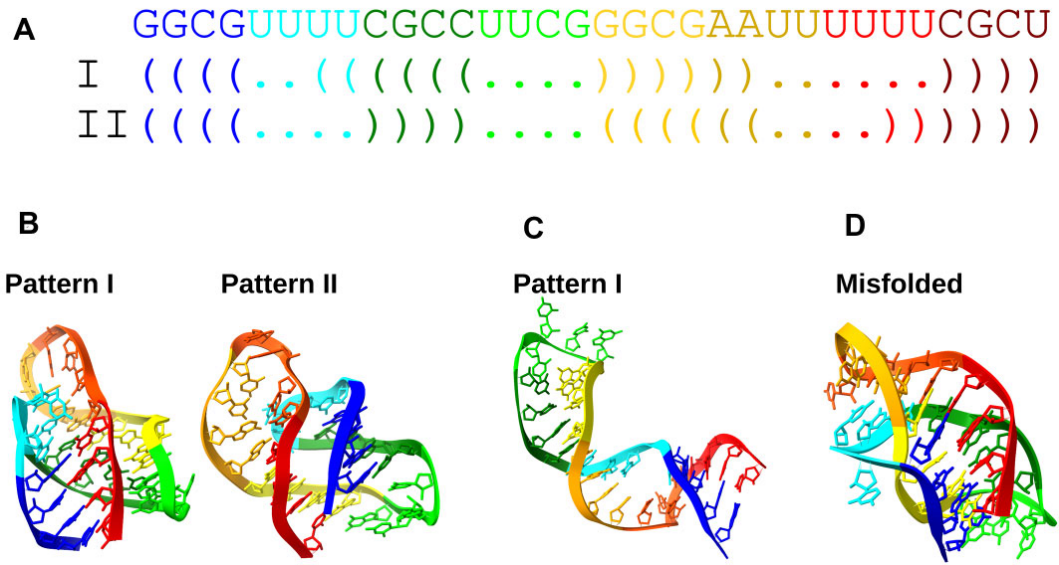
Demonstration of SimRNAweb v2.0 utility for folding alternative structures. (**A**) RNA sequence and its two alternative secondary structure patterns in a dot-bracket notation. (**B**) Results of folding with the new soft type of restraints: the first and fourth clusters correspond to the two alternative structures. (**C**) Results of folding without any restraints: only one of the alternative structures is obtained among top clusters of solutions. (**D**) Results of folding with the original hard type of restraints applied simultaneously for both structures: the resulting structure is misfolded.

Figure 1 illustrates the capabilities of the SimRNAweb v2.0 server in handling a short RNA sequence capable of forming two different, mutually exclusive secondary structures. Utilizing the advanced features of SimRNAweb v2.0, we explored the folding dynamics of this RNA sequence under different restraint conditions to showcase the server’s enhanced functionality. The input RNA sequence and its potential secondary structures were defined in dot-bracket notation, highlighting the sequence’s ability to fold into two alternative conformations. In the first simulation scenario, we employed the newly introduced soft restraints simultaneously for both alternative structures. This approach allowed the simulation to explore a wide conformational space, with a preference for stabilizing conformations containing either of the base pairs, without imposing strict penalties for the absence of these pairs. The results (Figure 1B) reveal that the first and fourth clusters of solutions indeed correspond to the two predefined alternative secondary structures. This outcome demonstrates the utility of soft restraints in guiding the folding process toward multiple potential conformations. When the folding simulation was conducted without any restraints, the server predominantly identified only one of the alternative structures among the top clusters of solutions (Figure 1C). This result underscores the challenge of capturing the full spectrum of potential RNA folds without specific guidance provided by restraints. Finally, a simulation incorporating the original hard type of restraints for both structures was performed. In this stringent setup, where both alternative structures were enforced simultaneously, the resulting structure was misfolded (Figure 1D). This misfolding arises from the inherent conflict between hard restraints attempting to enforce mutually exclusive conformations simultaneously, leading to an unrealistic and distorted RNA structure. These simulations collectively highlight the enhanced capability of SimRNAweb v2.0, which now offers a flexible and effective means of exploring the folding landscape of RNA sequences capable of adopting multiple structures. The ability to accurately model such complex folding behaviors is crucial for advancing our understanding of RNA structure and function.

#### Simulations with restraints on noncanonical base pairs

**Figure 2.**
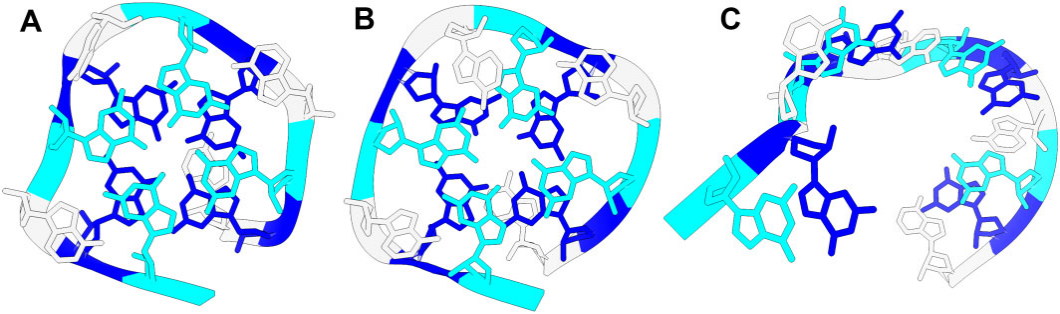
Demonstration of SimRNAweb v2.0 utility for the use of restraints on noncanonical base pairs. (**A**) Cartoon representation of an experimentally determined structure of an RNA aptamer against a bovine prion protein, which forms a G-quadruplex, devoid of any noncanonical pairs (RCSB PDB ID: 2RQJ; chain A). (**B**) Structure obtained via simulated annealing with hard restraints on noncanonical G–G WH *cis-*type base pairs. This is the first cluster representative with the best-scored structure, which shows excellent agreement with the experimentally determined structure [RMSD: 1.92 Å; Matthews correlation coefficient (MCC) for base pairs: 0.87]. (**C**) Structure obtained with the same parameters, but without restraints: due to the absence of canonical pairs, even low-energy conformations generated in the simulation without the restraints are largely unfolded, with the top cluster representative showing essentially no similarity to the reference structure (RMSD: 8.22 Å; MCC for base pairs: 0).

Figure 2 showcases the application of SimRNAweb v2.0 for incorporating restraints on noncanonical base pairs, specifically within the context of a G-quadruplex structure. This example highlights the server’s capability to accurately model complex RNA structures that include noncanonical interactions assigned by the user.

The folding simulations without any restraints resulted in misfolded structures. Despite numerous attempts, we were unable to obtain a correctly folded structure of this RNA with the current (3.22) version of SimRNA. Only the use of an experimental unpublished version of SimRNA with alternative energy parameters made such folding successful (data not shown). However, even with the current version of SimRNA, as implemented in SimRNAweb v2.0, we were able to reproduce the structure of the quadruplex, when the simulation utilized hard restraints on noncanonical G–G WH *cis-*type base pairs. The representative structure from the first cluster showed remarkable agreement with the experimentally determined structure, as evidenced by RMSD of 1.92 Å and MCC for base pairs of 0.85. These metrics indicate a high degree of structural similarity, underscoring the effectiveness of applying precise noncanonical pairing restraints in the modeling process. Through this example, SimRNAweb v2.0 demonstrates its advanced capability to incorporate noncanonical base pairing into RNA structure prediction.

#### Simulations with restraints from chemical probing data

**Figure 3.**
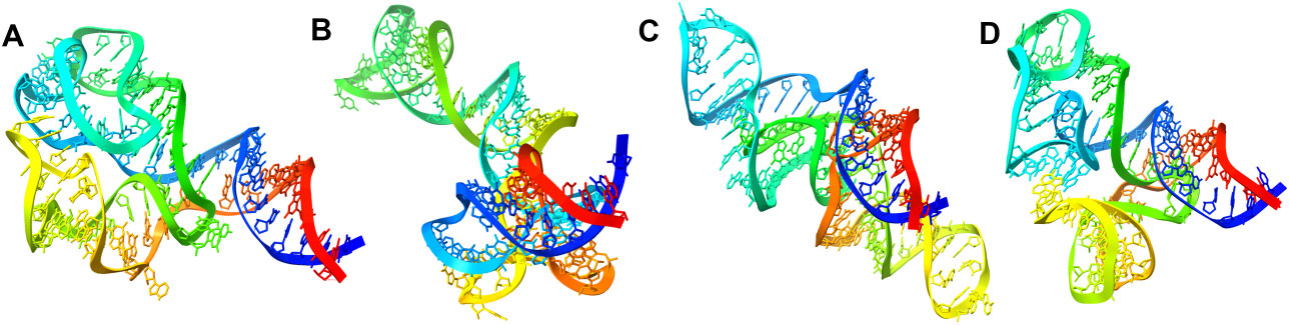
Demonstration of SimRNAweb v2.0 utility for the use of restraints derived from chemical probing data. (**A**) Cartoon representation of a structure of the *Escherichia coli* thiC riboswitch aptamer structure, modeled based on the experimentally determined structure of a closely related thiM riboswitch (RCSB PDB ID 4NYA). (**B**) Structure obtained via an REMC simulation without any restraints: this model is clearly misfolded (RMSD: 28.36 Å; MCC for base pairs: 0.29). (**C**) Structure obtained by an REMC simulation with the use of hard restraints on secondary structure: this model exhibits a correct secondary structure pattern, but the global 3D shape is not reproduced correctly (RMSD: 21.4 Å; MCC for base pairs: 0.64). (**D**) Structure obtained by an REMC simulation with the use of hard restraints on secondary structure as well as a pattern of residue reactivities from a SHAPE experiment (data generated in house, available on the web server as one of the examples): this model exhibits improved agreement with the experimentally determined reference 3D structure due to the formation of just a few critical long-range interactions (RMSD: 11.11 Å; MCC for base pairs: 0.65).

Figure 3 illustrates the effectiveness of SimRNAweb v2.0 in leveraging restraints derived from chemical probing data, specifically within the context of modeling the TPP riboswitch aptamer. This example underscores the server’s advanced capability to integrate experimental data into the RNA folding simulation process, enhancing the accuracy of predicted structures. In this case, a simulation conducted without any restraints fails to generate a correct structure. The inclusion of restraints on secondary structure did help to some extent, but the simulation failed to reproduce the global 3D shape of the riboswitch. This highlights the challenge of achieving precise three-dimensional structural fidelity through secondary structure restraints alone. Nonetheless, a simulation that utilized both secondary structure restraints and a pattern of residue reactivities derived from a SHAPE experiment demonstrated significantly improved agreement with the experimentally determined reference structure. The incorporation of chemical probing data provided a more nuanced and accurate representation of the riboswitch’s structural dynamics, and helped to model long-range interactions, enabling the simulation to more closely mimic the true 3D conformation. This example highlights the substantial benefits of integrating chemical probing data into RNA 3D structure prediction efforts.

## Discussion

The SimRNAweb server has been running since September 2015. It continues to provide an automated and user-friendly implementation of SimRNA, a method for RNA 3D structure modeling developed in our laboratory (3) and used in practice for modeling the structure of various RNAs, with the use of restraints derived from experimental data. One of many examples of our analyses is the modeling of the 5′-proximal regions in RNA genomes of betacoronaviruses, in which we used restraints derived from chemical probing experiments (12).

SimRNAweb server has been tested in benchmarking experiments, in particular in RNA-Puzzles (13,14), where it was used independently from the human-associated predictions. A prototypical version of the server was also tested by the GeneSilico group in the recent CASP experiment (15–17), however, only in the context of the human-assisted predictions. The updated version SimRNAweb v2.0 introduces new functionalities to the existing framework. The server now includes options for defining noncanonical base pairs in restraints, addressing an important aspect of RNA 3D structure. This feature facilitates the modeling of RNA structures that rely on non-Watson–Crick base pairs, such as G-quadruplexes. The addition of both hard and soft restraints enhances the server’s flexibility in modeling. Hard restraints retain the original functionality of enforcing the user-defined single structure, while soft restraints allow users to explore alternative RNA structures, useful in the study of molecules such as riboswitches that exhibit structural variability. The integration of the PDBx/mmCIF format in SimRNAweb v2.0 aligns with recent updates in data standards by RCSB PDB (18) improving the server’s compatibility with structural biology databases and software. The enhancements in this version extend its utility to a wider audience, including computational biologists and biophysicists, as well as experimental biologists interested in incorporating chemical probing data into RNA modeling.

With SimRNAweb v2.0, users can engage in a range of new applications. These include incorporating RNA chemical probing data into 3D structure prediction, conducting unfolding simulations for RNA thermal stability assessment and analyzing RNA conformational landscapes through clustering of trajectories with various parameters. These new features allow for a more detailed study of RNA structures, particularly those that exhibit alternative conformations.

The modeling with restraints presents certain limitations. The current input format for constraints on noncanonical base pairs necessitates that users specify the type of base pair. This functionality is especially beneficial for recognizing known RNA 3D motifs characterized by noncanonical interactions, such as k-turns, E-loops and others. G-quadruplexes, which rely on noncanonical base pairs where specific interactions in G-tetrads are identified, allow users to denote which residues pair and the type of base pair formed, as illustrated in the example discussed in this manuscript (Figure 2). However, predicting the specific type of noncanonical base pairs outside familiar motifs remains challenging. Most methods capable of predicting noncanonical pairs do so only at the residue pair level without specifying the exact type of noncanonical base pair. If the user is uncertain about the base pair type, the soft constraint feature permits the specification of alternative base-pairing possibilities for the same residue pair. SimRNAweb also gives the user the ability to impose restraints on the spatial proximity of the bases, without implying any particular type of the interaction. Examples have been added to the tutorial.

Regarding the interpretation of chemical probing data, SimRNAweb v2.0 employs a simplistic approach focused solely on base pairing, while the reactivity to chemical probes can be influenced by factors beyond the status of base pairing. Thus, on one hand, this method does not accommodate the inherent noise in chemical probing data, and on the other hand, it does not endeavor to interpret complex 3D structural features that may affect actual reactivity. Nonetheless, these types of restraints serve merely as a bias and do not drive the simulation to completely fulfill and potentially overfit the restraints. The current implementation enables users to input data that guide the simulation toward conformations enriched in regions of low reactivity in chemical probing experiments, which are preferably (but not exclusively) involved in forming helices or long-range interactions. The example provided in this manuscript (Figure 3) demonstrates that such restraints can enhance simulation outcomes beyond the application of conventional secondary structure restraints alone.

Future developments for SimRNAweb will focus on broadening its application scope and addressing the above-mentioned caveats. Planned updates include making available new versions and extensions of SimRNA, in particular versions with updated energy terms that facilitate the modeling of noncanonical pairs. These updates aim to keep SimRNAweb in step with the evolving needs of RNA biology and computational structure prediction research.

## Data Availability

The web server is available at https://genesilico.pl/SimRNAweb. This website is free and open to all users and there is no login requirement.
