# Correction to ‘SimRNAweb v2.0: a web server for RNA folding simulations and 3D structure modeling, with optional restraints and enhanced analysis of folding trajectories’

**DOI:** 10.1093/nar/gkae464

**Published:** 2024-05-27

This is a correction to: S Naeim Moafinejad, Belisa R H de Aquino, Michał J Boniecki, Iswarya P N Pandaranadar Jeyeram, Grigory Nikolaev, Marcin Magnus, Masoud Amiri Farsani, Nagendar Goud Badepally, Tomasz K Wirecki, Filip Stefaniak, Janusz M Bujnicki, SimRNAweb v2.0: a web server for RNA folding simulations and 3D structure modeling, with optional restraints and enhanced analysis of folding trajectories, *Nucleic Acids Research*, 2024; gkae356, https://doi.org/10.1093/nar/gkae356

In the originally published version of the article there is an error in the Funding statement. This should read: ‘Funding for open access charge: International Institute of Molecular and Cell Biology in Warsaw (IIMCB) funds.’

The published article has been updated.

